# Population dynamics of defective viral genomes of tomato black ring virus during host-to-host transmission

**DOI:** 10.1128/jvi.01244-24

**Published:** 2024-10-31

**Authors:** Daria Budzyńska, Julia Minicka, María J. Olmo-Uceda, Santiago F. Elena, Beata Hasiów-Jaroszewska

**Affiliations:** 1 Department of Virology and Bacteriology, Institute of Plant Protection-National Research Institute, Poznan, Poland; 2 Instituto de Biología Integrativa de Sistemas (I2SysBio), CSIC-Universitat de València, Valencia, Spain; 3 Santa Fe Institute, Santa Fe, New Mexico, USA; Iowa State University, Ames, Iowa, USA

**Keywords:** DVGs, host-to-host transmission, TBRV, virus experimental evolution

## Abstract

Defective viral genomes (DVGs) emerge during error-prone replication of viral genomes and contain deletions, insertions, genomic rearrangements, and hypermutations. These large-effect mutations result in the inability of DVGs to complete an infectious cycle in the absence of a helper wild-type virus. It has been shown that *in vitro* DVGs usually accumulate in viral populations when a virus is serially passaged in the same host at a high multiplicity of infection. To investigate the impact of host-to-host transmission on DVG formation and population dynamics *in vivo*, we conducted evolution experiments with tomato black ring virus (TBRV). TBRV was sequentially passaged through a combination of four distinct host species: quinoa, tobacco, lettuce, and spinach. The host was changed every fifth passage. The diversity and population dynamics of DVGs were analyzed based on the RNA-Seq data obtained through sequencing of viral RNA after 20 passages. Our findings indicate the possibility of TBRV DVGs generation when the virus was passaged through different host species. The level of DVG abundance varied across host plant combinations, with a weak indication that the host species past sequence may play a role in DVGs generation. Most abundant DVGs in the TBRV evolved populations were derived from RNA1. Deletions were the most prevalent class of DVGs, followed by insertions. The deletion DVG subpopulation exhibited substantial diversity in species composition and the richness of the deletions species was correlated with their abundance. Longer DVGs characterized by small deletions were predominant, whereas those shorter than 1,000 nucleotides constituted less than 2%.

**IMPORTANCE:**

Defective viral genomes (DVGs) have been identified *in vivo* and *in vitro* for different virus species infecting humans, animals, and plants. The ability to form DVGs during the passaging of virus in one host has been demonstrated, i.e., for tomato black ring virus (TBRV). In our research, RNA-Seq data obtained after TBRV passaging through a combination of four distinct host species were analyzed. Our results indicate that the level of DVG abundance varied across host plant combinations. Deletions were the most prevalent class of DVGs, with the domination of longer species. Additionally, the conserved junction sites in the TBRV genome were identified, resulting in the generation of identical deletions in independently evolved viral lineages. In summary, our findings provide significant insights into the origin and structure of DVGs of plant viruses. The obtained results will help in understanding viral evolution and host-virus interactions.

## INTRODUCTION

Defective viral genomes (DVGs) arose as a consequence of deletions, insertions, genomic rearrangements, and/or hypermutation that unavoidably appear as a consequence of the error-prone replication of RNA genomes (1). While DVGs exhibit high similarity to the genome of the parental virus, they are unable to self-replicate due to these defects. Their formation and propagation within the virus population depend on the presence of the wild-type parental helper virus. It is also suggested that DVGs retain signals essential for viral replication (2). Four predominant classes of DVGs can be distinguished: (i) deletions—generated when the viral polymerase skips part of the viral genome during the replication process, and the replication re-initiation site is on the same template, closer to the 5′ end; (ii) insertions—occur when re-initiation of the replication occurs at a position that is 3′ to the breakpoint; (iii) copy-backs; and (iv) snap-backs—generated when the viral polymerase detaches from template and continues copying the nascent strand (1–3).

Generation of DVGs applies to different virus species infecting humans (i.e., dengue virus, respiratory syncytial virus, SARS-CoV-2), animals (i.e., mouse hepatitis virus, vesicular stomatitis virus), or plants (i.e., tomato black ring virus), both *in vitro* and in nature (4–9). Although the main source of DVG formation is error-prone viral polymerase, the last decade of research concerning defective genomes revealed that a variety of factors may affect their generation, including host cellular components (10). Production and diversification of DVGs are favorable during the virus replication at high multiplicity of infection (MOI) (9). As DVGs can spread alongside complete viruses from one cell to another, they have the potential to modulate the infection outcome or aid in the persistence of the virus during infection (11). A particularly important feature of some DVGs is their ability to trigger an antiviral immune response and interfere with virus replication (7, 12). These defective interfering particles (DIPs or DI RNAs) are being considered potential components for antiviral therapies and vaccines (13).

Previous research has suggested that DVGs undergo continuous and radical changes, including additional point mutations, insertions, deletions, and recombinations/rearrangements similar to what occurs to the parental virus. Furthermore, it is hypothesized that due to competition with the parental virus for replication resources, DVGs are under strong selective pressure and can evolve faster than the parental virus (14–16).

Virus evolution is a change in the genetic structure of viral populations over time leading to the emergence of new variants, strains, or species possessing unique biological properties (17–20). It is driven through genetic drift and selection, imposed by factors such as vectors, environmental conditions, or host species and genotypes. For plant viruses, these evolutionary forces often are associated with transmission between different host plants (21). During host-to-host transmission, viruses must interact and adapt to the host’s defense systems, accumulating mutations that may enhance or diminish their fitness or infectivity in different hosts (18, 20, 22, 23). Host switching challenges viruses with strong adaptive selection pressure to maximize their fitness in the new environment and may speed up evolutionary dynamics (18, 21, 24). There are two hypotheses regarding the dependencies between virus virulence and the ability for between-host transmission. The first assumes that virulence reduces the between-host transmission, leading to selection pressures that act to maximize transmission by reducing virulence (25). The second hypothesis posits trade-offs between virulence and transmission: high levels of replication may increase the probability of a pathogen being transferred to a new host, but this could also result in rapid host mortality, limiting the window for transmission (25). In addition to point mutations, recombination plays a pivotal role in virus evolution and epidemiology, which has been associated with the emergence of new viral variants/species, expansion of viral host ranges, the alteration of transmission vector specificities, and increases in virulence and pathogenesis. It has been suggested that those viruses that recombine at the highest frequencies are more prone to crossing species boundaries (26).

There are numerous examples of host-to-host transmission in plant viruses resulting in the generation of DVGs, including DI RNAs. The ability to form defective particles during passaging of the virus in one host has been demonstrated, i.e., for tomato black ring virus (TBRV) (6–8, 27). TBRV (species *Nepovirus nigranuli*, genus *Nepovirus*, family *Secoviridae*) is a widespread pathogen infecting economically important plant species including vegetables and fruits, ornamental species, or perennial plants. The genome of the virus consists of two positive-sense ssRNAs of about ~7,400 and ~4,600 nucleotides in length (28). Additionally, the TBRV genome can be accompanied by nonstandard viral genomes like DI RNAs and satellite RNAs (satRNAs). Both DI RNAs and satRNAs significantly affect viral load (7, 29). It has been found that (i) DI RNAs of TBRV might arise during prolonged passages in *Chenopodium quinoa* or *Nicotiana tabacum*; (ii) DI RNAs derive from parental virus as a result of single deletion in RNA1 or RNA2; (iii) DI RNAs are about ~270–600 nucleotides in length; and (iv) DI RNAs affect virus transmission through seeds and interfere with TBRV replication (6–8, 27, 30).

To examine the impact of host-to-host transmission on the formation of DVGs and their population dynamics, we conducted evolution experiments involving the passaging of a TBRV isolate through different sets of host species. A total of 20 passages were performed, during which the TBRV isolate was sequentially transferred through four distinct host species combinations consisting of different host plants: lettuce (*Lactuca sativa*, family *Asteraceae*), quinoa (*C. quinoa*, subfamily *Chenopodioideae*, family *Amaranthaceae*), spinach (*Spinacia oleracea*, subfamily *Chenopodioideae*, family *Amaranthaceae*), and tobacco (*N. tabacum*, family *Solanaceae*). Each combination comprises three independent evolutionary lineages, and the host changes occurred following every five passages. Subsequently, after 20 passages, RNA-Seq analysis was conducted, and the presence of DVGs and the structure of the DVGs population were analyzed using different bioinformatic tools.

## RESULTS

### TBRV DVGs are generated *de novo*

To evaluate the DVG composition in each evolved lineage after the 20th passage of evolution, we analyzed the results from DVGfinder (ran in consensus mode) obtained for the ancestral virus (hereafter referred to as TBRV-Anc) and for the 12 evolved lineages.

TBRV-Anc isolate was obtained through agroinoculation of test plants with a previously obtained cDNA infectious clone (31). Notice that during the preparation of inoculum (multiplication and purification of the virus), the virus was transferred between quinoa plants three times. Thus, it is expected that the parental virus isolate contains a certain amount of DVGs, which arose during virus replication in quinoa.

Table 1 shows an approximated estimate of virus accumulation based on the reads mapping to the reference genome. We tested the hypothesis that the production of DVGs is directly proportional to viral replication, i.e., no replicative advantage exists for the DVGs. Under this hypothesis, a positive correlation between these two variables is expected. By contrast, if DVG replication occurs independently of the quantity of viral genomes, no correlation is expected. Interestingly, a nonsignificant correlation was found (Spearman partial correlation controlling for the scenarios: *ρ*_PS_ = 0.012, 10 d.f., *P* = 0.971), which suggests that the production of DVGs has a negative, though minor, effect of virus accumulation.

**TABLE 1:** Total number of mapped reads into the viral genome (expressed as RPHT), as a proxy to viral accumulation and abundance of DVGs [presented as a sum of mean RPHT (mean of reads per hundred thousand obtained with DI-tector and ViReMA)] in each evolution lineage and TBRV-Anc^*a*^

Scenario	Lineage		Accumulated mean RPHT				
		Virus load^*b*^	All DVGs	Deletion	Insertion	5´cb/sb	3´cb/sb
	TBRV-Anc	49,497.964	138.539	106.773	31.156	0.009	0.601
S1	L1	43,402.571	187.977	115.573	47.245	15.154	10.004
	L2	41,311.885	316.467	225.902	59.393	12.193	18.979
	L3	37,741.925	198.481	138.812	38.883	15.030	5.755
S2	L4	49,876.559	91.873	30.187	42.061	13.604	6.021
	L5	49,891.069	78.250	26.557	35.449	11.829	4.415
	L6	44,831.168	531.169	490.709	25.353	10.231	4.875
S3	L7	49,955.966	592.918	558.121	20.997	9.761	4.039
	L8	49,953.765	671.708	654.373	8.517	6.909	1.909
	L9	48,364.101	545.071	488.928	38.083	13.519	4.540
S4	L10	49,987.980	71.983	21.541	34.160	11.658	4.624
	L11	43,334.064	91.493	56.586	21.171	10.884	2.852
	L12	49,843.161	59.478	25.248	22.339	9.266	2.625

^*a*^ Accumulated mean RPHT values were rounded to three decimal digits.

^*b*^ Reads mapped to the reference genome relative to the library size (VRPHT).

**Fig 1:**
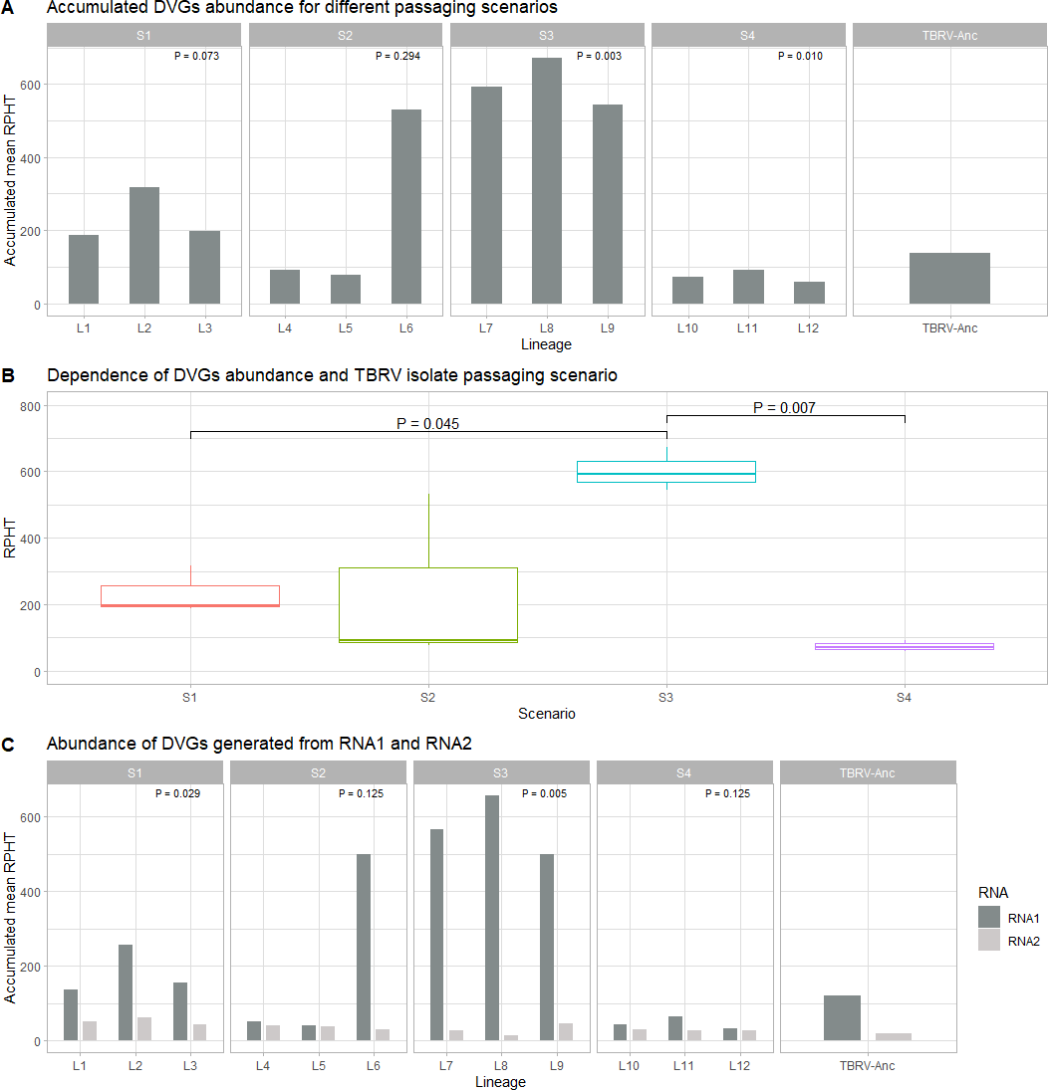
Abundance of DVGs in each evolved lineage. (**A**) Comparison of the abundance of DVGs observed for each host-switching scenario and in the TBRV-Anc population. *P* values represent the result of one-sample *t*-test comparing the mean of the corresponding three lineages with the TBRV-Anc observed value. (**B**) The same data as in (**A**) but compared across the four scenarios. *P* values represent Tukey HSD *post hoc* tests. (**C**) Accumulated mean RPHT (reads per hundred thousand) data from (**A**) segregating by genomic RNA1 and RNA2. *P* values correspond to paired-sample *t*-tests.

The abundance of all DVG types in TBRV-Anc was compared with the results from each lineage (L1-L12) and presented in Table 1. Reads of different types of DVGs were detected in all samples, with some lineages clearly showing an increase in DVGs content relative to the ancestral virus, while other lineages showed a decrease (Fig. 1A). At the one side, the largest increase was found for lineages evolved under scenario S3 (mean DVG reads per hundred thousand viral reads, RPHT = 592.918, 671.708, and 545.071 for L7, L8, and L9, respectively, one-sample *t*-test, *P* = 0.003). On the other side, significant decreases were observed for lineages evolved at S4 (mean RPHT = 71.983, 91.493, and 59.478 for L10, L11, and L12, respectively, one sample *t*-test, *P* = 0.010). While the abundance of all DVGs in three lineages evolving at scenarios S1, S3, and S4 were relatively similar, for S2, the accumulated RPHT of DVGs in L6 (531.169) largely deviated from values obtained for two other lineages (91.873 and 78.250 for L4 and L5, respectively).

Overall differences in DVGs abundance across the four scenarios were found (Fig. 1B; Welch’s robust one-way ANOVA, *P* = 0.002). Indeed, the effect was largely explained by the significant differences between scenarios S1-S3 and S3-S4 (Fig. 1B; Tukey HSD *post hoc* tests, *P* = 0.045 and *P* = 0.007, respectively). This result suggested further exploring whether the differences in DVG content among scenarios could be explained by differences in the complexity of the temporal sequences of succession of the four host species. First, as a measure of temporal complexity, we computed the type-token ratio (TTR) (32). According to this statistic, S1 was the simplest scenario (TTR = 0.2), followed by S2 and S4 (TTR = 0.35), and S3 was the most complex one (TTR = 0.4). No significant correlation exists between the scenario’s temporal complexity and the observed abundance of DVGs (Spearman correlation, *ρ*_S_ = 0.316, 2 d.f., *P* = 0.684). Second, to test whether similarity among scenarios would result in more similar DVG profiles, we computed the sequence matcher distance (SMD) (33). Scenarios S2 and S4 are the most similar ones (SMD = 0.322), while S2 and S3 are the most dissimilar ones (SMD = 0.661). A marginally significant correlation exists between this distance and the median difference in DVG abundance among scenarios (*ρ*_S_ = 0.794, 4 d.f., 1-tailed *P* = 0.029), suggesting that lineages evolved under more similar host switching histories tend to show more similar DVG abundances.

A common pattern observed across lineages was that DVGs originated from RNA1 were significantly more abundant than those from RNA2 (Fig. 1C; Wilcoxon paired-samples test, *P* < 0.001); the magnitude of the difference depending on the specific scenarios, and particularly significant in the case of S1 and S3 (Fig. 1C; paired samples *t*-tests, *P* = 0.029 and *P* = 0.005, respectively).

### Prevalence of DVG classes

**Fig 2:**
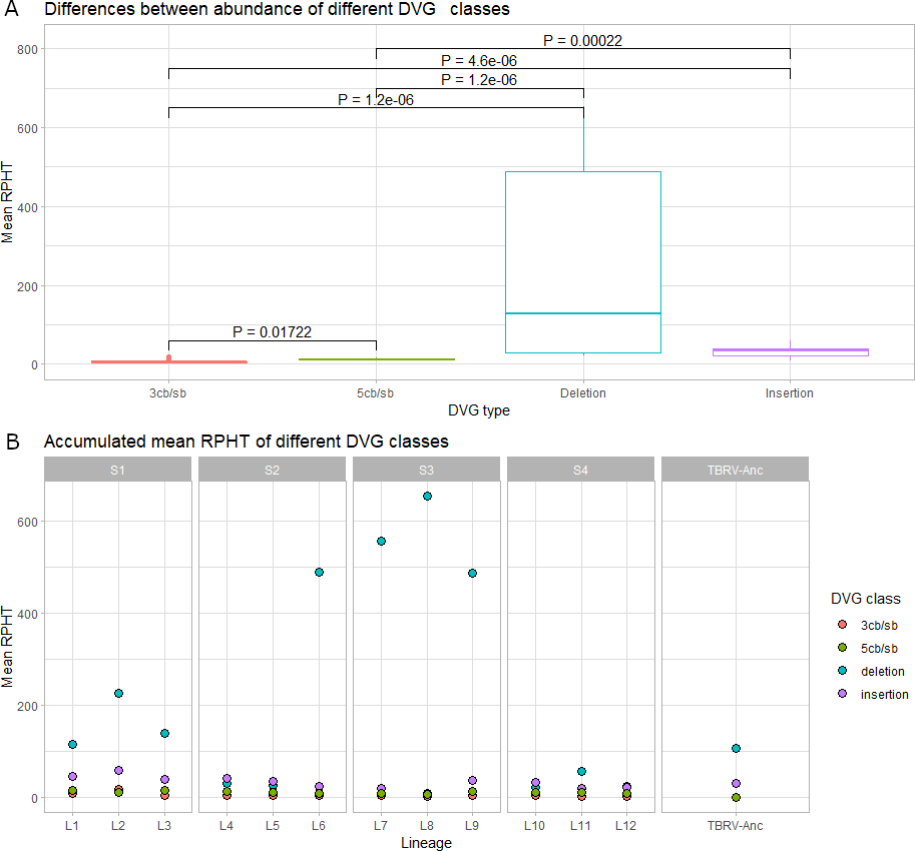
Analysis of the abundance of different classes of DVGs. (**A**) Differences between the abundance of four different DVG classes. (**B**) The same data as in (**A**) but segregated by scenarios and lineages. The values estimated for TBRV-Anc are also shown for comparison.

Deletions, insertions, and copy-back/snap-back (cb/sb) DVGs were identified in all lineages (Table 1) although with significantly different abundances (Fig. 2A; Kruskal-Wallis test, *P* < 0.001). Overall, deletions were the most abundant class, followed by insertions and 5′ cb/sb at approximately the same amount as 3′ cb/sb (Fig. 2A). However, if lineages are analyzed independently, L4 and L5 in S2 and L10 in S4 do not show a dominance of deletions (Fig. 2B).

### Diversity of the deletion DVGs population

Due to the large number of detected DVGs of all classes and previous literature reports concerning the biological relevance of TBRV deletion, the following analyses will solely focus on the deletion class of DVGs (6–8, 27).

**Fig 3:**
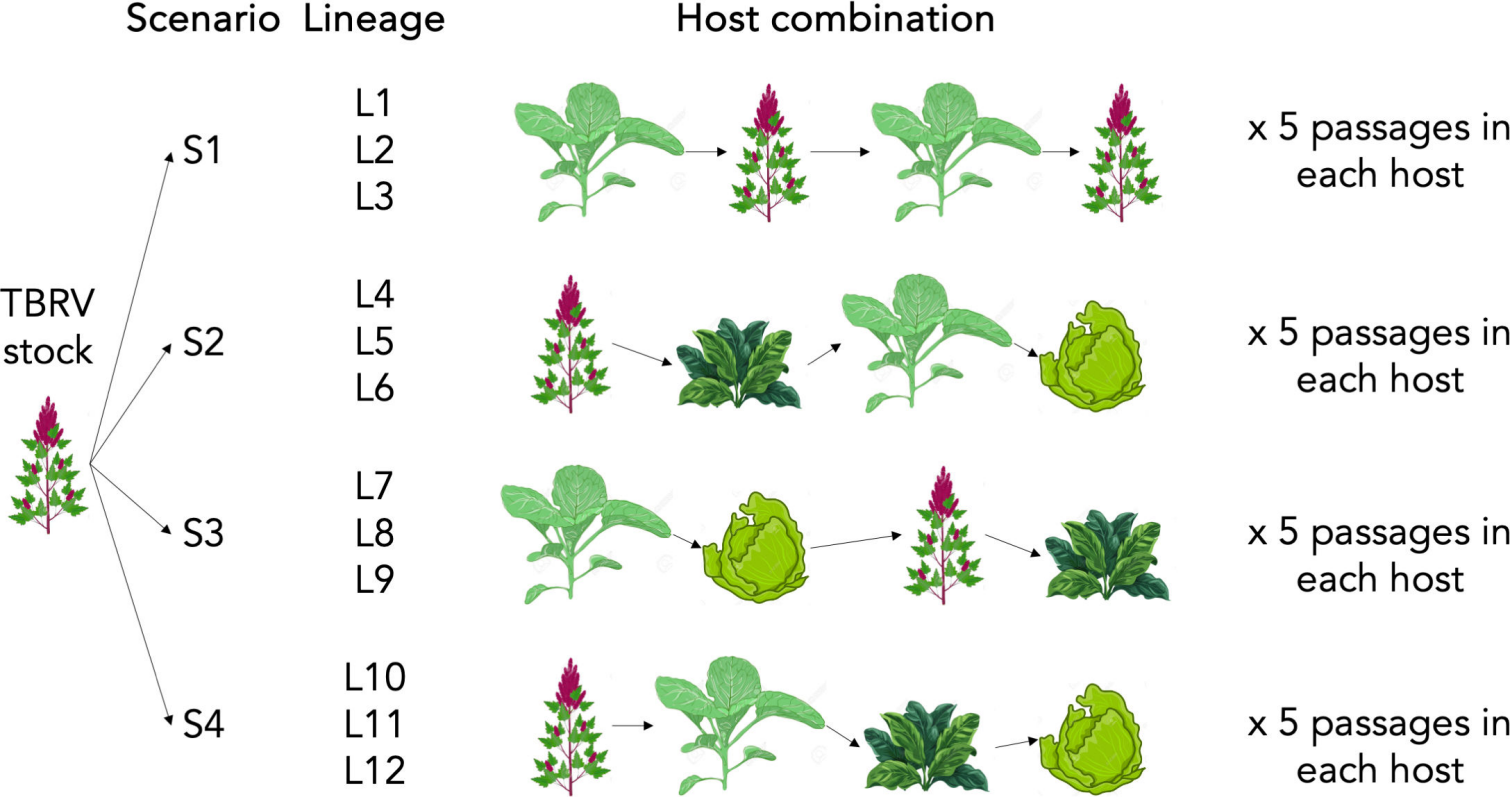
Schematic representation of the evolution experiment. In each host TBRV isolate were passages five times, after the fifth passage host changing occurred. Host plant combinations were assigned as passaging scenarios S1–S4. In each scenario, virus was passaged in three independent lineages.

The diversity of the deletion DVGs subpopulation was evaluated as the number of unique DVG species found in each lineage. DVG diversity varied among lineages (Table 2). The most diverse deletion subpopulation was found in the TBRV-Anc population (4,191 unique DVG species). After evolution under the different host switches scenarios (Fig. 3), the diversity of deletion subpopulations decreased, varying between 648 (L8) and 1,986 (L1) across lineages (Table 2). RNA1 and RNA2 significantly differ in terms of generating unique deletions, with RNA1 being significantly more prone to generate DVGs of different species than RNA2 (Wilcoxon signed ranks test, *P* < 0.001). In L10, the number of unique DVGs generated from RNA1 was over 17.4-fold higher than those produced from RNA2. Despite these differences between lineages, no overall differences among scenarios have been in terms of classes of deletions neither when considering the whole genome (one-way Welch’s robust ANOVA, *P* = 0.283) nor RNA1 and RNA2 separately (one-way Welch’s robust ANOVA, *P* = 0.156 and *P* = 0.525, respectively).

**TABLE 2:** Diversity of the deletion DVGs subpopulation (number of unique DVG types) in each evolved lineage and TBRV-Anc

Lineage	Number of unique deletion species			Percent of DVGs with mean read counts ≥5	Percent of DVGs longer than 1,000 nt
	RNA1	RNA2	Total		
TBRV-Anc	3,171	1,020	4,191	1.36	99.62
L1	1,638	348	1,986	14.45	98.69
L2	1,420	247	1,667	18.96	99.34
L3	977	179	1,156	14.71	98.96
L4	1,743	195	1,938	13.16	99.58
L5	1,319	152	1,471	12.64	98.84
L6	726	190	916	16.70	99.45
L7	698	216	914	14.88	98.58
L8	497	151	648	16.67	98.92
L9	870	320	1,190	14.62	99.24
L10	1,441	83	1,524	11.22	99.34
L11	699	145	844	13.98	99.53
L12	1,351	241	1,592	12.69	99.12

**Fig 4:**
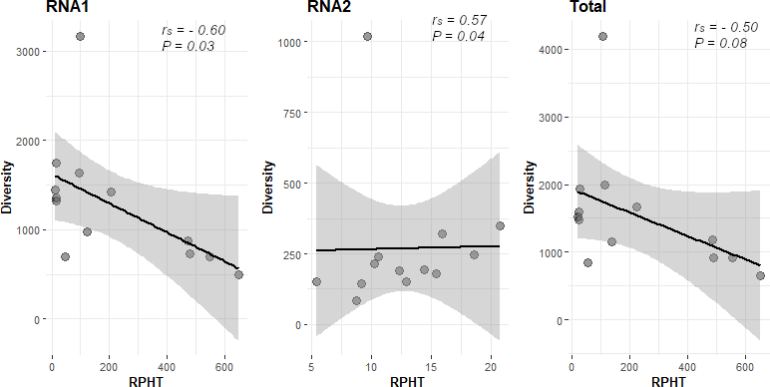
Relationship between DVG abundance (total number of counts) and diversity (number of different species). The association was evaluated for each genomic RNA as well as for the whole genome. Spearman’s correlation coefficients and their corresponding *P* values are reported in each case. Solid lines correspond to the linear regression model and gray shadow to its 95% confidence interval.

Next, the dependence between the number of unique deletions and their abundance was considered. Under the hypothesis of selective pressures acting on particular DVGs, it is expected that an increase in selected DVGs abundance would reduce the differentiation of DVGs species among populations. To test this hypothesis, a correlation analysis was performed between the accumulated mean RPHT of all deletion DVGs and their number of species, as well as for those generated from RNA1 and RNA2 of TBRV (Fig. 4). The hypothesized negative correlation was confirmed in the case of RNA1 (*ρ*_S_ = −0.599, 11 d.f., *P* = 0.034).

These differences in DVGs diversity could be explained by different selective pressures acting on the DVG species. For the TBRV-Anc isolate, from among 4,191 different DVGs species, the presence of only 1.36% was confirmed with at least five reads (mean reads aligned with two algorithms), whereas for lineages L1–L12, this value varies between 11.22% (L10) and 18.96% (L2) (Table 2).

### Long deletion DVGs are the most abundant

For both, the TBRV-Anc isolate and the L1–L12 evolved lineages, longer deletions species were the most pervasive, with DVGs with a length above 1,000 nucleotides constituting more than 98.58% of all detected deletions (Table 2). Among the deletion DVG species analyzed for TBRV-Anc and each lineage separately, the DVG labeled as 7125_7128 [numbers denote breaking point (BP) and rejoining point (RI) coordinates, respectively] generated from RNA1 was highly prevalent, in 11 out of 13 samples (Table 3). In the two lineages, L4 and L10, in which this deletion was not observed, the most prevalent one was 6300_6303, in both cases. Among DVGs generated from RNA2, the most abundant was 3116_3177, present in the TBRV-Anc and all evolved lineages.

**TABLE 3:** Most abundant deletion DVG species in different samples^*a*^

Lineage		ID	Length	Mean read counts	Mean RPHT
TBRV-Anc	RNA1	7125_7128	7,381	17,303	75.23
	RNA2	3116_3177	4,613	538	2.55
L1	RNA1	7125_7128	7,381	35,435	76.20
	RNA2	3116_3177	4,613	1,614	8.46
L2	RNA1	7125_7128	7,381	77,743	186.06
	RNA2	3116_3177	4,613	632	4.55
L3	RNA1	7125_7128	7,381	35,760.5	109.86
	RNA2	3116_3177	4,613	654.5	5.63
L4	RNA1	6300_6303	7,381	201	0.39
	RNA2	3116_3177	4,613	876.5	6.01
L5	RNA1	7125_7128	7,381	526.5	1.08
	RNA2	3116_3177	4,613	683.5	5.12
L6	RNA1	7125_7128	7,381	172,197.5	468
	RNA2	3116_3177	4,613	972.5	5.37
L7	RNA1	7125_7128	7,381	224,802	538.53
	RNA2	3116_3177	4,613	655	3.09
L8	RNA1	7125_7128	7,381	324,226	642.68
	RNA2	3116_3177	4,613	316.5	1.24
L9	RNA1	7125_7128	7,381	162,351	460.82
	RNA2	3116_3177	4,613	1,284	6.07
L10	RNA1	6300_6303	7,381	201	0.40
	RNA2	3116_3177	4,613	420	4.46
L11	RNA1	7125_7128	7,381	15,274	38.52
	RNA2	3116_3177	4,613	552.5	3.33
L12	RNA1	7125_7128	7,381	2,004	3.52
	RNA2	3116_3177	4,613	943	3.54

^*a*^ ID of the DVGs includes information about BP and RI sites.

The abundance of the prevalent deletion RNA1 7125_7128 varied among lineages: from a minimum of 1.08 in L5 to a maximum of 642.68 in L8 (Table 3). Interestingly, the abundance of this long deletion had increased relative to the TBRV-Anc isolate in seven lineages (L5, L11, and L12 being the exception). For these lineages, the overall abundance of DVGs as well as an abundance of deletion DVGs was lower in comparison to other samples. For deletion RNA2 3116_3177, its abundance had also increased in all lineages, except in L8, compared to TBRV-Anc (Table 3).

### Insights into RNA1 and RNA2 error-prone regions and lineage-specific variations

**Fig 5:**
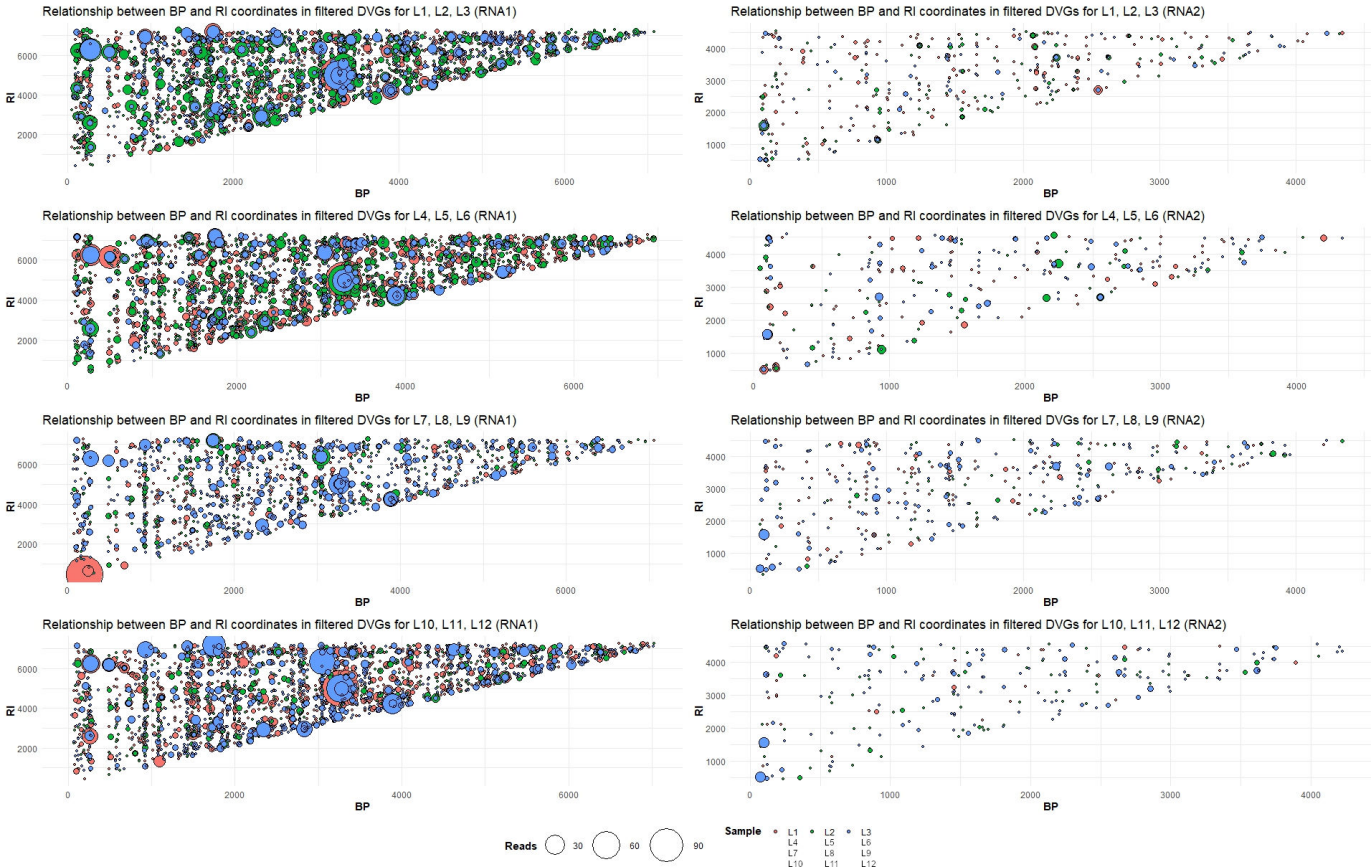
Distribution of BP and RI coordinate of DVGs arose in lineages evolved in the four different scenarios for RNA1 (left) and RNA2 (right). The size of the dots represents the number of mean read counts. Different dot colors represents particular lineages (orange: L1, L4, L7, L10; green: L2, L5, L8, L11, blue: L3, L6, L9, L12).

First, given the large number of detected DVGs, DVGfinder results were filtered to retain only deletions of at least 100 nucleotides. As illustrated in Fig. 5, RNA1 BP/RI positions are distributed along the entire sequence. However, most of them were detected with only a few reads, constituting a background noise from which recombination hotspots pop up. Although the number of deletion species generated from RNA2 is lower, some predominant species can also be distinguished (Fig. 5). For both RNA1 and RNA2, prevalent deletion species found across lineages have been observed (Fig. 5). We hypothesized that there are specific hotspot positions in the TBRV genome playing a role in polymerase dissociation and replication re-initiation, resulting in the accumulation of the most abundant DVGs, regardless of the host-switching scenario in which the lineages evolved. To test this hypothesis, we filtered previously analyzed data (mean RPHT >0.018) and analyzed deletion distribution across genomic RNA.

Based on these results, a few conclusions can be made: (i) RNA1 is more recombination-prone than RNA2; (ii) some of the deletions found both in RNA1 and RNA2 of TBRV-Anc are pervasively observed (leading to the formation of different length DVGs with common BP or RI coordinates), most of them do not include the 5′ and 3′ end of RNA1 or RNA2 (Fig. 5). The region of genomic RNA, which is subjected to deletion mainly covers nucleotides position between ~1,480 and ~7,030 in RNA1 and positions between ~243 and ~4,295 in RNA2; (iii) some deletions observed in TBRV-Anc population were lost in all evolved L1–L12 lineages. Despite the distribution of deletions size and abundance differ among lineages, a few of them seem to be preserved in the TBRV population in most of them (region between ~3,240 nt and ~4,985 of RNA1, Fig. 5). Finally, (iv) the coverage of the deletions in the virus populations coming from lineages seems not to be scenario-specific but shared by some of the lineages (Fig. 5).

### Some of the deletion DVGs are pervasive in different virus populations

Based on previous literature reports concerning TBRV DVGs (specified as defective particles), we hypothesized that the generation of some DVG species is favored by the existence of recombination hotspots and those DVGs would be produced regardless of the scenario of host switching under which virus evolved. As previously, only DVGs with deletions covering ≥100 nucleotides were considered. An RPHT >0.018 was set as a threshold for persistence. To avoid false negatives, in the case of identifying a given deletion DVG in only one lineage, the three lineages evolved under the same scenario, or most (10–12) lineages, the presence of such DVG was also confirmed in unfiltered data of the other lineages and in TBRV-Anc.

**Fig 6:**
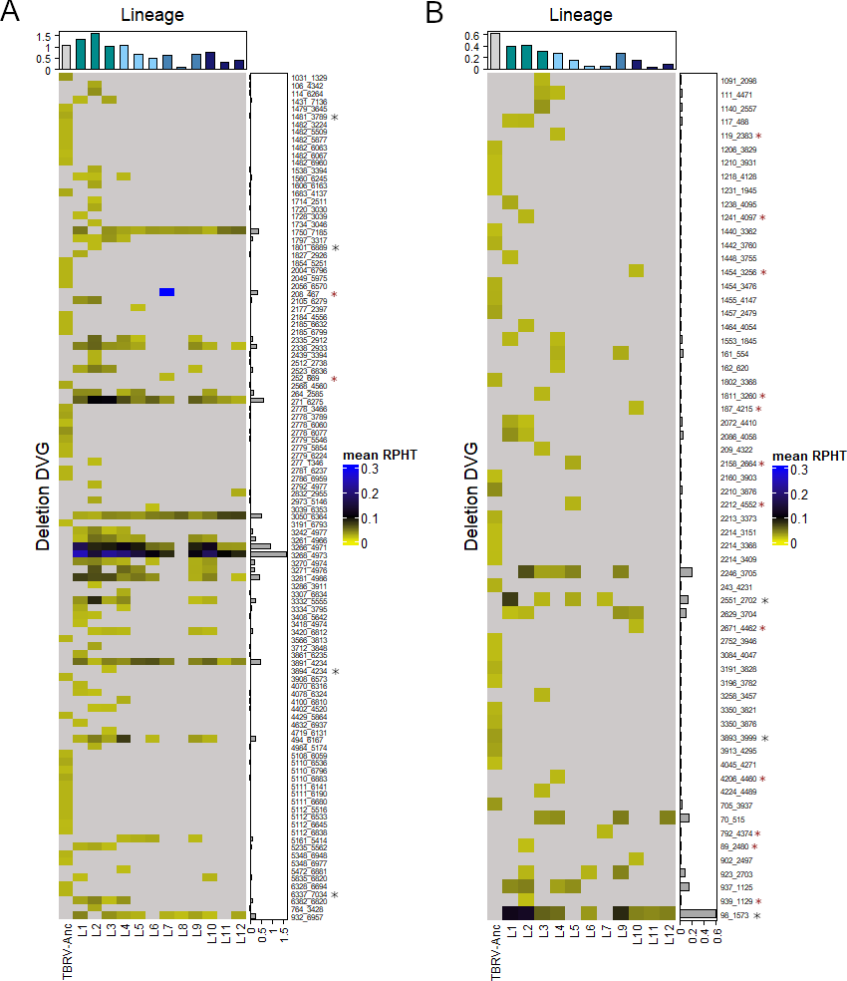
Heatmap illustrating the presence of DVGs with deletions longer than 99 nucleotides and mean RPHT > 0.018. (**A**) For RNA1. (**B**) For RNA2. Upper barplots represent the accumulated RPHT per lineage, and side barplots the accumulated RPHT of each DVG. Black asterisks indicate DVGs also found in TBRV-Anc, while red asterisks mark those present in only one lineage.

Out of all identified deletions, only deletion RNA1 3050_6364 has been found in all 12 evolved lineages (Fig. 6A; mean RPHT from 0.025 to 0.071), and the overall differences in this DVG abundance were affected by the host switching scenario under which the virus evolved (one-way ANOVA, *P* = 0.023). Significant differences in mean abundance were found for scenarios S1 and S4 (Tukey HSD *post hoc* test, *P* = 0.018). Moreover, this particular deletion seems to be generated *de novo*, during passages, since it was not present in the TBRV-Anc population. After further investigations, several DVGs shown in Fig. 6 were identified in all 12 lineages. However, in every case, the abundance of at least one of them was below the RPHT = 0.018 threshold (i.e., 271_6275, and 3266_4971). For DVGs generated from RNA2, no example of DVG pervasive across the 12 lineages was found (Fig. 6B). For RNA1, the most abundant deletion species was 3268_4973 (mean RPHT = 1.644), whereas for RNA2, it was 98_1573 (mean RPHT = 0.516).

Some of the deletion DVGs were unique for a certain lineage. For example, RNA1 deletions 252_669 and 208_467 were only found in L7 (Fig. 6A). By contrast, lineage-exclusive deletions generated from RNA2 were more frequent: 12 DVGs were assigned as exclusive to one lineage (Fig. 6B). None of the DVGs described above was present in the TBRV-Anc population, which suggests that they arose *de novo*, during the passages. *De novo* formation of deletions seems to be a common feature for TBRV; as from all the most abundant DVGs presented in Fig. 5, only four DVGs generated from RNA1 (1481_3789, 1801_6889, 3894_4234, and 6337_7034) and three generated from RNA2 (2551_2702, 3893_3999, and 98_1573) were already present in TBRV-Anc (mean RPHT < 0.018).

Previously, *in vivo* identified DI particles from RNA1 had BP/RI 185_6830, 206_6941, 147_6862, 147_6840, 371_7322, and 371_7243 (RNA1), while from RNA2, the only DI previously described had BP/RI 318_4488 (6–8, 27). Although the analyses here presented do not recover any of these precise BP/RI, the presence of several DVGs with nearby junction sites has been found in RNA1 (e.g., 184_6827, 249_6843, 241_6860, and 209_6863).

## DISCUSSION

Until the end of the 20th century, numerous defective viral particles from different viruses were identified. However, due to the difficulties with their analyses, the usage of the terms “defective particles” and “defective interfering particles” was mostly limited to a narrow group of short RNAs generated from the parental virus genome that may have had a phenotypic effect in virus accumulation or symptoms. The development of high-throughput sequencing methods (HTS) has allowed unprecedented detail in analyzing recombination and DVGs, showing that the subpopulation of incomplete viral genomes is more diverse and pervasive than was previously assumed.

Accumulation of DVGs is presumably a combined result of their *de novo* generation from the wild-type virus genome followed by consecutive replication and incorporation of DVGs into the viral particle. *In vitro,* DVGs are usually generated when the virus is serially passaged in the same host (31, 34, 35). In this study, we showed that both, expansion of pre-existing DVGs and *de novo* generation of additional ones, are possible when the virus is passaged to different host species. Moreover, significant differences in overall DVGs abundance between lineages evolving under different host switching scenarios (sets of consecutive host plants) were observed, suggesting an impact of the past host species on DVGs accumulation. Interestingly, we found that the complexity of the scenarios in terms of plant species succession had no measurable effect on the composition of the DVG subpopulation. In contrast, we found a positive yet marginally significant association between the similarity of scenarios and their DVG composition. These differences occurred between lineages passaged through scenarios S3 and S4 and scenarios S1 and S3. In the first case, composition of the plant species in each set was the same, which may justify, that among the pool of the same host plants, the temporal order of hosts plays a crucial role in DVGs generation. It has been previously shown that TBRV accumulation strongly depends on host species which was higher in quinoa and tobacco in comparison to spinach and lettuce (7, 29). Influence of MOI on successful DVGs has been also previously confirmed (9, 36). In this study, the mean abundance of all DVGs for S3 lineages (RPHT = 603.25) is over eight times higher than for S4 lineages (RPHT = 74.318). In scenario S3, the virus was passaged alternating host species most preferred by the virus, which might result in higher viral accumulation and formation of DVGs. In scenario S4, the host species in which virus accumulation was lower were placed at the end of the passaging experiment, which might decrease the virus accumulation, and, therefore, affected DVGs abundance. On the other hand, in scenarios S2 and S4, both ending in lettuce, the overall DVGs abundance is indistinguishable. Highlighting the possibility that that only the final host defines the DVGs abundance. This possibility deserves further investigation.

In the TBRV population, DVGs generated from RNA1 are the most abundant, and the most unbalanced proportion of RNA1/RNA2 DVGs was observed for L8, with ~45 times more DVGs from RNA1 than from RNA 2 (RPHT = 655.31 and 14.40, respectively). TBRV RNA1 encodes proteins necessary for virus replication including the viral polymerase. The relatively low abundance of DVGs produced from non-polymerase segments was confirmed for the influenza virus which could be related to the localization of structural elements required for polymerase binding and/or replication (37).

Among all DVG classes, the most abundant were deletions followed by insertions, which were predominant in lineages L4, L5, and L10. The prevalence of the deletions has been confirmed for the other positive-stranded RNA virus species (35, 38). Taking into account the previous findings concerned TBRV DVGs, deletion was expected as a predominant class in our experiments (7, 8, 27).

The population of TBRV DVGs is diverse in terms of DVGs species composition (unique DVG types). The most diverse seemed to be DVGs population of TBRV-Anc, where 4,191 unique DVGs species were identified, whereas for L1–L12, the number varied between 648 and 1,986. A negative correlation between the diversity of deletion DVGs and their overall abundance was observed although it depended on the particular RNA1 and RNA2. It has been hypothesized that continuous competition takes place between different DVG species coexisting in the same infected host, and then the best competitors are positively selected (1). Selection of the DVGs is complex and may be affected by their length and nucleotide composition and regulated by other viral and host factors (10).

Interestingly, we observed that for TBRV the predominant deletion DVG subpopulation was composed of long DVGs species (from 1,000 to 7,381 nucleotides), while those shorter than 1,000 nucleotides constituted less than 2% of all deletions detected. According to the previously formulated competition hypothesis, shorter DVGs should dominate owing to their faster replication. However, this hypothesis needs to be further explored and carefully tested since a bias for shorter sizes could be due to the preferential generation of short DVG and/or maybe simply a spurious result due to the limitation of detection methods like RT-PCR. The limitation of RT-PCR is mainly due to the necessity of using specific primers and the method’s sensitivity. The use of HTS methods led us to conclude that the junction sites leading to the DVGs formation are localized across the whole genome, and the diversity of TBRV DVGs is much higher than it has been previously assumed. Recent data have shown that the predominance of longer DVGs in the viral population takes place when the virus replicates in an environment that limits virus replication (9).

According to our previous findings that short DVGs of TBRV interfere with virus replication (7), we decided to narrow down our analysis to DVGs containing deletions of at least 100 nucleotides. We hypothesized that there are specific positions in the TBRV genome, favoring polymerase dissociation and replication re-initiation, resulting in persistent DVGs across different lineages. Regions in the TBRV genome which are prone to be deleted mainly cover the fragment between ~1,480 and ~7,030 in RNA1 and positions between ~243 and 4,295 in RNA2 and do not include 5′ and 3′ ends of both RNAs. These noncoding sequences of nepoviruses contain conserved sequence motifs, which likely play a role in the replication or translation of viral RNAs (39). It is also hypothesized that the C-terminus of the replicase of two other *Nepovirus* members, grapevine fanleaf virus and arabis mosaic virus, is predicted to be a potential host range determinant (40).

Among the deletion DVGs identified within the different viral lineages, we identified a few which were shared by all 12 lineages, with DVG 3050_6364 being the most abundant. We did not identify any DVG that showed a host-switching scenario specificity. Also, some of the DVGs found exclusively in one lineage were produced as a result of deletion in RNA2. Most of the low-frequency DVGs described arose *de novo*, as we did not find evidence of their presence in the TBRV-Anc DVGs population. However, deletions RNA1 7125_7128 and RNA2 3116_3177 were already present in the TBRV-Anc. These two deletions were found in almost all lineages at higher abundance, suggesting they may play some beneficial effect for the viral population as a whole or have a fitness advantage in competition with other DVGs. These two possibilities deserve future investigation.

To close our study, we investigated in all DVGs subpopulations the presence of DVGs types previously described for the natural TBRV infections. One theory concerning the generation of shorter DVGs is that those particles are produced as a result of the continuous shortening of the premature forms (41). Although we did not find DVGs with the same BP and RI coordinates, the presence of longer DVGs with junction sites nearby those previously described in the literature (6–8, 27) was found.

## MATERIALS AND METHODS

### Plant material

The previously obtained infectious cDNA clone of TBRV-P1 isolate was used for agroinfiltration of tobacco (cv. Xanthi) plants (31). Agroinoculated plants were maintained under greenhouse conditions (22–23°C, 16 h light/8 h dark photoperiod). After 14 days post inoculation (dpi), 100 mg of apical leaves was harvested and used for total RNA isolation with RNeasy Plant Mini Kit (Qiagen). The presence of the virus in infected plants was confirmed by RT-PCR with previously obtained RNA and specific primers amplifying the viral *cp* gene (42).

Subsequently, virus-positive plants were used for inoculation of quinoa plants, and purified virus preparations were obtained in sucrose gradient as described previously (27). Viral RNA was isolated using the phenol-chloroform method, and RNA profile of the TBRV isolate was analyzed in agarose gel.

### Evolution passages, RNA preparation, and sequencing

The obtained viral RNA was diluted to 1 µg/µL and used to inoculate three quinoa plants. After 7 dpi, 500 mg of TBRV-infected plants were ground in a phosphate buffer (0.05 M, pH 7.2) and used as an inoculum in the evolution experiment. The passages were conducted in four different combinations of host plants (hereafter referred as scenarios, Fig. 3) with three independent evolving lineages: (i) tobacco, quinoa, tobacco, quinoa (scenario S1, lineages L1–L3); (ii) quinoa, spinach, tobacco, lettuce (scenario S2, lineages L4–L6); (iii) tobacco, lettuce, quinoa, spinach (scenario S3, lineages L7–L9); (iv) quinoa, tobacco, spinach, lettuce (scenario S4, lineages L10–L12). The change of host plant was carried out after five passages in each particular species (Fig. 3; e.g*.*, in scenario S2: five passages in quinoa → five in spinach → five in tobacco → five in lettuce). Passages were performed at a 7-days interval. After 7 dpi, 500 mg of each TBRV-infected plant was ground in a phosphate buffer (0.05 M; pH 7.2) and used to inoculate new, virus-free plants. Two of four host species used in the experiment, tobacco and quinoa, were previously used to generate TBRV DVGs after serial passages (6–8, 27) and are known as permissive hosts for TBRV replication. The other two plant species, lettuce and spinach, are known as experimental hosts for TBRV (7, 29). Notice that quinoa and spinach are taxonomically closely related species (within the subfamily *Chenopodioideae*), while all four species are only related at the level of class *Eudicots*.

The complexity of the different scenarios was quantified using the type-token ratio (TTR) method (32) as the number of instances in each scenario in which a given host-to-host transition (e.g., quinoa—tobacco or quinoa—quinoa) appeared only once, divided by 20, the total number of transition events. The method was implemented in the R package “text” version 1.2.1. Similarity among scenarios was computed using the sequence matcher distance (SMD) (33) that measures the similarity between two scenarios by finding the longest contiguous matching subsequence of hosts. The ratio of the lengths of these matching subsequences to the total length of the strings (i.e., 20) gives a measure of their similarity. The method was implemented in the R package “stringdist” version 0.9.5.0. R version 4.4.0 was run in RStudio version 2024.04.2+764.

After the 20th passage, from each lineage, the virus was purified in a sucrose gradient, and viral RNAs were isolated using a phenol-chloroform method and treated with Turbo DNase (Invitrogen) according to the manufacturer’s instruction. The quality of particular RNAs was checked with NanoDrop 2000 (Thermo Fisher Scientific), Qubit 3 Fluorometer (Thermo Fisher Scientific), and agarose gel electrophoresis. Libraries were prepared with NEBNext Ultra II RNA Library Prep Kit for Illumina (New England Biolabs)/VAHTS Universal V6 RNA-seq Library Prep Kit for Illumina (Vazyme Biotech) by Genomed S.A. (Warsaw, Poland) or weSEQ.it (Rybnik, Poland), and then samples were sequenced using the NovaSeq 6000 platform with 150 nt paired-end technology, resulting in ~42–81 millions of reads per sample.

### RNA-Seq data analysis

The quality check of obtained RNA-Seq data was performed with FastQC (43) and MultiQC (44) tools. Adapter-trimming, quality-trimming, and filtering of the paired reads were done using BBDuk.sh (https://sourceforge.net/projects/bbmap/); as a result, good quality cleaned fastq files were obtained.

Cleaned fastq files of ancestor TBRV-P1 isolate were mapped to TBRV-P1 genome (GenBank, acc. no. KX977560.1, KX977561.1) with CLC genomic Workbench. The consensus sequences were extracted as fasta files and used in further analyses. The total number of reads mapping into the reference genome, after discounting reads mapping into more than one site, was used as a proxy to viral accumulation and expressed as reads per hundred thousand of total reads (VRPHT = 10^5^·viral reads/total reads).

The presence of the DVGs in the resulting high-throughput sequencing data was assessed with DVGfinder (45). As an input paired-end reads fastq files interleaved with BBmap (https://sourceforge.net/projects/bbmap/) and previously obtained consensus ancestor TBRV-P1 genomic sequences in fasta format as well as files indexed with bwa were used (46).

Next, the obtained results were filtered according to the DVG type, deletion size, and number of reads. We focused only on DVGs generated as a result of forward/reverse deletion in the TBRV genome, as this type of DVG has been previously described for the virus. Breakpoints (BP) and rejoining sites (RI) predicted by DVGfinder were used to calculate the size of the deleted genome fragment during the DVG formation. Only those DVGs that were indicated by both algorithms were used for further analyses.

The quantity of DVGs was computed as accumulated mean of reads per hundred thousand (RPHT = 10^5^·DVG reads/viral reads). In other words, the mean number of reads where the BP/RI junction has been found relative to the total number of reads mapped to reference sequence per 10^5^ (45).

## Data Availability

Raw RNA-Seq data have been deposited to NCBI SRA (BioProject accession number PRJNA1135462).
